# Soft-Tissue-Mimicking Using Hydrogels for the Development of Phantoms

**DOI:** 10.3390/gels8010040

**Published:** 2022-01-06

**Authors:** Aitor Tejo-Otero, Felip Fenollosa-Artés, Isabel Achaerandio, Sergi Rey-Vinolas, Irene Buj-Corral, Miguel Ángel Mateos-Timoneda, Elisabeth Engel

**Affiliations:** 1 Centre CIM, Universitat Politècnica de Catalunya (CIM UPC), Carrer de Llorens i Artigas, 12, 08028 Barcelona, Spain; ffenollosa@cimupc.org; 2 Departament of Mechanical Engineering, Barcelona School of Industrial Engineering (ETSEIB), Universitat Politècnica de Catalunya, Av. Diagonal, 647, 08028 Barcelona, Spain; irene.buj@upc.edu; 3 Department d’Enginyeria Agroalimentària i Biotecnologia, Escola d’Enginyeria Agroalimentària i de Biosistemes de Barcelona, Universitat Politècnica de Catalunya, Carrer Esteve Terradas, 8, 08860 Barcelona, Spain; maria.isabel.achaerandio@upc.edu; 4 Institute for Bioengineering of Catalonia (IBEC), The Barcelona Institute of Science and Technology (BIST), c/Baldiri Reixac 15-21, 08028 Barcelona, Spain; srey@ibecbarcelona.eu (S.R.-V.); mamateos@ibecbarcelona.eu (M.Á.M.-T.); eengel@ibecbarcelona.eu (E.E.); 5 Bioengineering Institute of Technology, Universitat Internacional de Catalunya (UIC), c/Josep Trueta s/n, 08195 Barcelona, Spain; 6 CIBER en Bioingeniería, Biomateriales y Nanotecnología (CIBER-BBN), 28029 Madrid, Spain; 7 IMEM-BRT Group, Departament de Ciència i Enginyeria de Materials, Universitat Politècnica de Catalunya, Av. Eduard Maristany 10-14, 08019 Barcelona, Spain

**Keywords:** dynamic mechanical analysis, hardness, hydrogels, materials, mimicking, soft tissues, tissue scaffolding, viscoelasticity, Warner–Braztler shear test

## Abstract

With the currently available materials and technologies it is difficult to mimic the mechanical properties of soft living tissues. Additionally, another significant problem is the lack of information about the mechanical properties of these tissues. Alternatively, the use of phantoms offers a promising solution to simulate biological bodies. For this reason, to advance in the state-of-the-art a wide range of organs (e.g., liver, heart, kidney as well as brain) and hydrogels (e.g., agarose, polyvinyl alcohol –PVA–, Phytagel –PHY– and methacrylate gelatine –GelMA–) were tested regarding their mechanical properties. For that, viscoelastic behavior, hardness, as well as a non-linear elastic mechanical response were measured. It was seen that there was a significant difference among the results for the different mentioned soft tissues. Some of them appear to be more elastic than viscous as well as being softer or harder. With all this information in mind, a correlation between the mechanical properties of the organs and the different materials was performed. The next conclusions were drawn: (1) to mimic the liver, the best material is 1% wt agarose; (2) to mimic the heart, the best material is 2% wt agarose; (3) to mimic the kidney, the best material is 4% wt GelMA; and (4) to mimic the brain, the best materials are 4% wt GelMA and 1% wt agarose. Neither PVA nor PHY was selected to mimic any of the studied tissues.

## 1. Introduction

Hydrogels are hydrophilic gels, polymer networks that are swollen with water as the dispersion medium. They are an excellent solution for different medical applications such as bone regeneration [1,2], tissue engineering [3,4] or, soft-tissue-mimicking [5].

Regarding the latter application, studying the mechanical characterization of real soft tissues is an important approach to understanding how they are deformed during different clinical scenarios such as surgeries. In this way, different solutions could be developed to reduce soft tissue damage. Nonetheless, using real biological tissues can be difficult for two reasons: (1) accessibility, and (2) ethics [6]. In this way, a possible solution could be to use surgical planning prototypes, also known as phantoms, which are simulated biological bodies.

Surgeons only have a short period to carry out complex technical tasks during the operation. Therefore, it could be vital to know in advance what exactly must be done, during surgery to shorten the operation duration and thereby reduce surgical-related risks. However, to date doctors have not received enough training and methods to face this problem [7]. Additionally, different studies demonstrated that surgeons who trained with physical models had better skills in comparison with those who did not have the same opportunity [8].

Considering this, it is necessary to find materials that mimic the properties of biological tissues. Until now, different materials such as silicones, hydrogels, or photopolymer resins were studied [6,9,10,11,12,13,14,15,16,17,18,19,20,21,22,23]: PVA, PHY, agar, gelatine, alginate, hydrogels or Sylgard 527 (PDMS) and Sylgard 184 (Silicone Elastomer), as well as photopolymer resins for Additive Manufacturing (e.g., VeroWhite+, a rigid general purpose, high resolution, opaque white material; and TangoPlus+, which simulates thermoplastic elastomers with flexible, rubber-like qualities; both of them developed by Stratasys^®^). Additionally, it is worth mentioning that there are other biopolymeric materials such as aerogels that can be used for tissue engineering and regenerative medicine [24,25]. For example, Yahya et al. [24] highlighted the main challenges of biopolymer-based scaffolds and the prospects of using these materials in regenerative medicine.

Of these materials, hydrogels (hydrophilic water-based gels [26]) were mainly used in different studies; yet they only focused on one or two organs. For example, Forte et al. [18] and Leibinger et al. [17] mimicked the brain tissue by developing a composite hydrogel (6% wt PVA/0.85% wt PHY [18] and 5% wt PVA/0.59% wt PHY [17]). Additionally, Tan et al. [6] mimicked three soft living tissues (brain, lung, and liver) by molding in a petri dish using different compositions of PVA and PHY. It was determined that the best compositions for each organ were the following: (1) to mimic the brain, 2.5% wt PVA + 1.2% wt PHY; (2) to mimic the lung, 11% wt PVA; and (3) to mimic the liver, 14% wt PVA + 2% wt PHY. This study was the first important step for the realization of surgical planning training devices and tools. Dister et al. [23] measured similar viscoelastic properties of the brain tissue by developing a 0.5% wt alginate/0.5% wt gelatine. Adams et al. [9] used the molding technique for obtaining different soft kidney phantoms using silicone elastomer, agarose gel, or PDMS.

Among these studies, not all of them covered in-depth viscoelastic properties as well as the Shore hardness of the materials. These two properties are important parameters that are normally measured in soft-tissue-biomechanics.

Regarding the viscoelastic properties, few studies covered the viscoelasticity of soft tissues in recent years. For instance, Chatelin et al. [27] reviewed the viscoelastic properties of the brain tissue studied during the last 50 years. Then, Estermann et al. [28] studied the viscoelasticity of the liver tissue using DMA. It was found that at a frequency of 1 Hz the storage and loss modulus measured at porcine and bovine liver were extremely high. For example, the storage and loss modulus for the porcine is 488.3 ± 163.9 kPa and 52.23 ± 28.91 kPa, respectively. Other studies [29,30,31], on the other hand, showed lower values in the range of 0.5–9 kPa and 0.07–1 kPa for the storage and loss modulus, respectively. Focusing on the kidney, as it happened with the liver, some discrepancy was seen [32,33]. For example, Charles [32] measured high values: for E′, around 40 kPa; and for E″, around 17 kPa whereas Amador [33] got lower values for storage and loss modulus: 4.5–5.5 kPa (for η = 10 Pa·s) and 4.5–9 kPa (for η = 100 Pa·s) and 0.026–1.08 kPa (for η = 10 Pa·s) and 0.25–2.2 kPa (for η = 100 Pa·s), respectively. The heart [34] has a storage modulus (E′), which varies from 20 KPa at 0.5 Hz to 40 KPa at 3.5 Hz. On the other hand, the loss modulus (E″) values are between 3 kPa at 0.5 Hz and 8 kPa at 3.5 Hz.

On the other hand, another important parameter is hardness, which can be measured either by indentation or abrasion. For the soft biological tissues, macroindentation has been previously used to measure the organs’ hardness. The hardness is a tactile property, which is useful by manual palpation to distinguish healthy from pathological tissues [22]. In this way, it is an important parameter to take into consideration. There are various types of hardness tests for hard materials such as Vickers (HV), Brinell (HB), or Rockwell (HR). Regarding the characterization of the soft tissues, Shore hardness is the most commonly used [5,13,22,35,36,37]. As an example of this, Yoon et al. [35] measured a 15.06 ± 2.64 Shore 00 in a healthy human liver. Additionally, Estermann et al. [22] measured a 30.52 ± 1.52 Shore 00 for a porcine liver, whereas for a bovine liver was 25.67 ± 2.61 Shore 00. Additionally, with the Shore hardness test, the shear stiffness of the soft tissue can be obtained as described in [38]. Regarding the pancreas, Belyaev et al. [13] and Foitzik et al. [36] measured a 20 Shore 00 and 26.3 ± 2.5 Shore 00, respectively. Then, Riedle et al. [37] obtained different values, even harder: for the arcus aortae, 13.4 ± 1.9 Shore A; for the thoracic aorta, 17.1 ± 1.4 Shore A; and for the aorta abdominalis, 17.6 ± 1.2 Shore A.

Additionally, from the authors’ knowledge, less research has been carried out studying the cut feeling (related to shear strength), a crucial factor to be considered during surgical procedures [39]. Quantifying this property can be vital in the tissue-mimicking field to achieve more realistic phantoms. Warner-Bratzer (WB) shear test is used widely in food science to evaluate meat or animal muscle tenderness. The test is considered as an empirical method; however, some authors have found a statistical correlation between the maximum tensile force (mechanical property) and WB shear force in beef muscle, related to the strength of the muscle fibers [40].

Overall, the present paper aims to address several soft living tissues mimicking using different hydrogels. For that, the measurement of the viscoelastic properties will be carried out using the DMA (Dynamic Mechanical Analysis) technique and the hardness using a Shore Hardness durometer as well as the cut feeling concept by carrying out the Warner–Bratzler shear test. In this way, the mimicking between six hydrogels (based on agarose, PVA, PHY, and GelMA) and four soft living tissues (liver, heart, kidney, and brain) can be achieved. The reason for the large extent of organs and materials, which is a novelty in this research field, is both the wide range of values detected in previous articles and the intention to be able to use the mimicking data obtained to address, in a future stage, the 3D printing of multi-material prototypes. Therefore, this study offers originality not only because the Warner–Bratzler Shear test can be used as a new mimicking method, but also due to the wide range of soft living tissues and materials that are analyzed.

## 2. Results and Discussion

In this study, we aimed to mimic several soft living tissues with different materials taking into consideration several parameters: (1) viscoelastic properties using compression tests (E′ and E″); (2) Shore hardness; and (3) the “cut feeling” concept by carrying out the Warner–Bratzler shear test, which measured the forces (shear strength) profile along the cut. The materials that are out of range of the soft-tissue-biomechanics are not included in the different plots.

### 2.1. Liver

**Figure 1:**
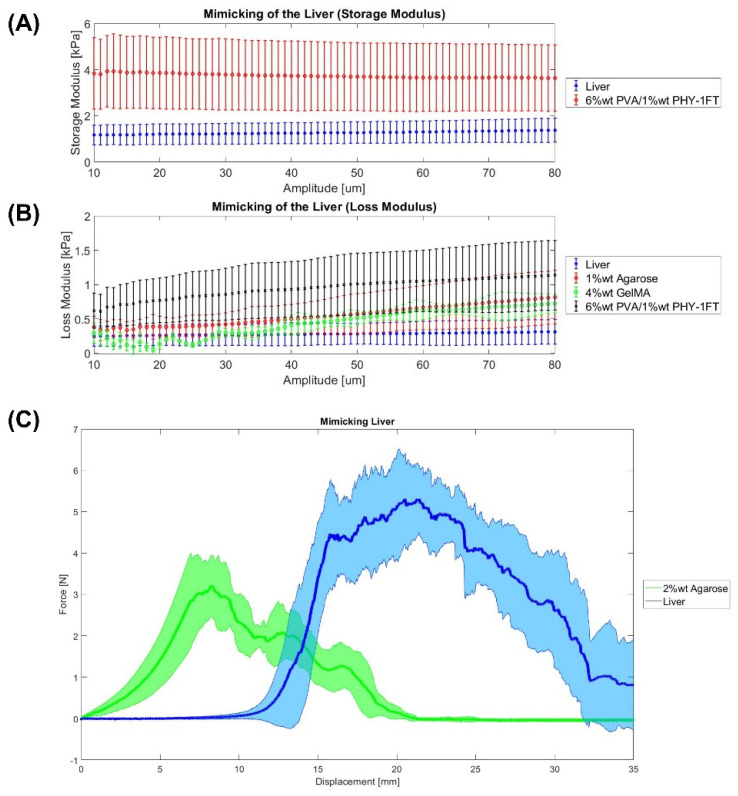
Mimicking the liver. (**A**,**B**) DMA results. (**C**) Warner–Bratzler shear test results. Data were represented as mean ± SEM values. Each sample has an *n* = 6.

Firstly, the liver Shore hardness obtained in our experiments is 13 ± 4.5 Shore 00, which has been measured as is described in Section 4.3.2. This value is similar to the result obtained by Yoon et al. [35], which is 15.06 ± 2.64 Shore 00 in a healthy liver. Additionally, Estermann et al. [22] measured a 30.52 ± 1.52 Shore 00 for a porcine liver, whereas for a bovine liver was 25.67 ± 2.61 Shore 00. Higher numbers on the scale indicate a greater resistance to indentation and, hence, harder materials. In this study, the material which matched more closely the hardness of the liver tissue is 1% wt agarose gel (14 ± 2.5 Shore 00). Regarding this material, Oflaz et al. [13] obtained a value of 27.25 ± 2.72 Shore 00 for 1% wt agarose gel. Secondly, this study measured a liver tissue storage and loss modulus of 1.2 ± 0.5 kPa and 0.27 ± 0.17 kPa, respectively. These results were similar to other studies [29,30,31] which showed values in the range of 0.5–9 kPa and 0.07–1 kPa for the storage and loss modulus, respectively. On the other hand, Estermann et al. [28] also studied the viscoelasticity of the liver tissue. However, it was found that at a frequency of 1 Hz the storage and loss modulus measured at porcine and bovine liver were extremely high, which is not common for soft tissues. For example, the storage and loss modulus for the porcine liver is 488.3 ± 163.9 kPa and 52.23 ± 28.91 kPa, respectively [28]. Then, Figure 1 shows the best materials for mimicking the viscoelastic properties of the liver. In both storage and loss modulus, the 6% wt PVA/1% wt PHY-1FT hydrogel that undergoes one freeze-thaw cycle seemed to be the best option. This composite hydrogel was also used with similar PVA and PHY amounts by Tan et al. [6]. In terms of the Warner–Bratzler shear test, Figure 1C shows the liver tissue cutting profile. The peaks observed might be related to the presence of internal blood vessels as are veins and arteries. Additionally, it might also be because it is a heterogeneous organ. Regarding the mimicking, among all materials, no hydrogel was able to match its cutting profile. Finally, it is important to highlight the mimicking achieved in other studies. For example, de Jong et al. investigated that a 4% wt PVA hydrogel that undergoes two freeze-thaw cycles can mimic the liver tissue. For instance, Tan et al. [6] studied a composition hydrogel of 14% wt PVA/2.1% wt PHY that undergoes one freeze-thaw cycle was determined to mimic the liver.

A statistical analysis was carried out to clarify the effectiveness of the present tissue-mimicking analysis. Table 1 shows the statistical analysis of the liver and the closest materials using the *t*-test. If the *p*-value is higher than 0.05, the null hypothesis stated before is not rejected and the material matches the organ.

### 2.2. Heart

**Figure 2:**
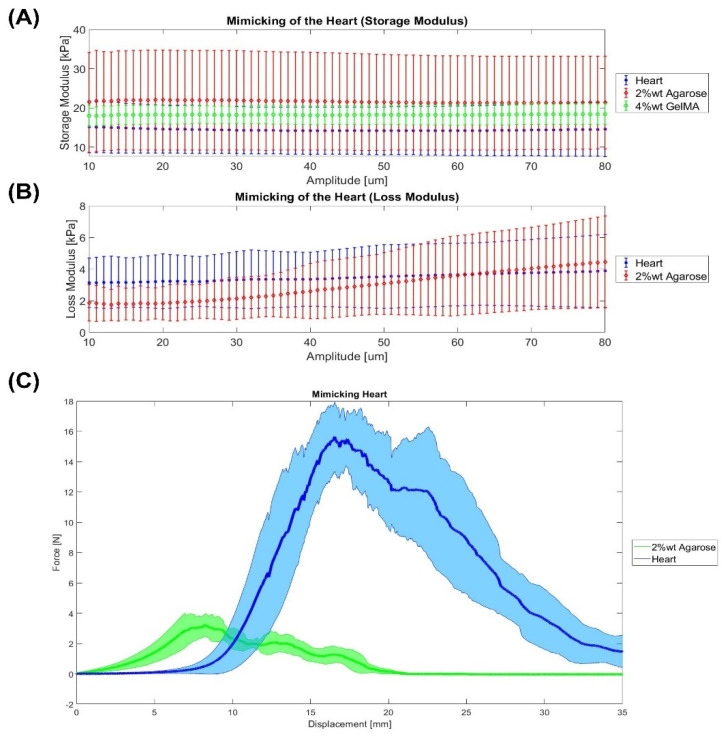
Mimicking the heart. (**A**,**B**) DMA results. (**C**) Warner–Bratzler shear test results. Data were represented as mean ± SEM values. Each sample has an *n* = 6.

Firstly, the heart Shore hardness is 20 ± 7.5 Shore 00, which means that it is harder than the liver. This might be because the heart is a muscle. Riedle et al. [38] obtained different values, even harder: for the arcus aortae, 13.4 ± 1.9 Shore A; for the thoracic aorta, 17.1 ± 1.4 Shore A; and for the aorta abdominals, 17.6 ± 1.2 Shore A. In this study, the material which matched more closely the hardness of the heart tissue is 1% wt agarose gel (14 ± 2.5 Shore 00). Secondly, this study measured a heart tissue storage and loss modulus of 14.5 ± 5.5 kPa and 3.40 ± 1.87 kPa, respectively. Regarding the storage modulus, the values of our study are lower than the values measured by Ramadan et al. [34] from 20 kPa at 0.5 Hz to 40 kPa at 3.5 Hz. On the other hand, the loss modulus (E″) values are between 3 kPa at 0.5 Hz and 8 kPa at 3.5 Hz, which are close to our results. In addition, the stiffness lies from 400 to 800 N/m with a phase shift around 0.175. In our study, the phase shift is 0.22, which means that a more vicious behavior was obtained. Then, Figure 2A,B show the best materials for mimicking the viscoelastic properties of the heart. In both storage and loss modulus, 2% wt agarose gel seemed to be the best material for mimicking the viscoelastic behavior of the heart. In terms of the Warner–Bratzler shear test, Figure 2C shows the heart tissue seems to have a straight slope at the beginning of the cutting profile since the samples used did not take into consideration the possible holes in which the blood flows. Additionally, it is interesting to see that the maximum is higher than some of the other soft tissues, which might due to the presence of the cardiac muscles. Regarding the “cut feeling” mimicking, 2% wt agarose is the material that more closely resembles the mechanical properties of native heart tissue. Although as can be seen in Figure 2C, it is still far from the heart tissue. This means a higher amount of agarose would be needed. In the literature, however, different materials have been used for the mimicking of the heart tissue. For instance, Yoo et al. [41] used the material jetting technology for 3D printing the heart. For that, the most flexible material (TangoPlus FullCure resin) for the heart, a solid material (VeroWhite) for the platform and stools, and a mixture of the 2 print materials for valvar annuli were used. In another study, Riedle et al. [42] 3D printed in red translucent silicone with a Shore A hardness of 20 (ACEO^®^ Silicone GP Shore A 20, Wacker Chemie AG) using the ACEO^®^-technology.

A statistical analysis was carried out to clarify the effectiveness of the present tissue-mimicking analysis. Table 2 shows the statistical analysis of the heart and the closest materials using the *t*-test. If the *p*-value is higher than 0.05, the null hypothesis stated before is not rejected and this means that the material matches the real organ.

### 2.3. Kidney

**Figure 3:**
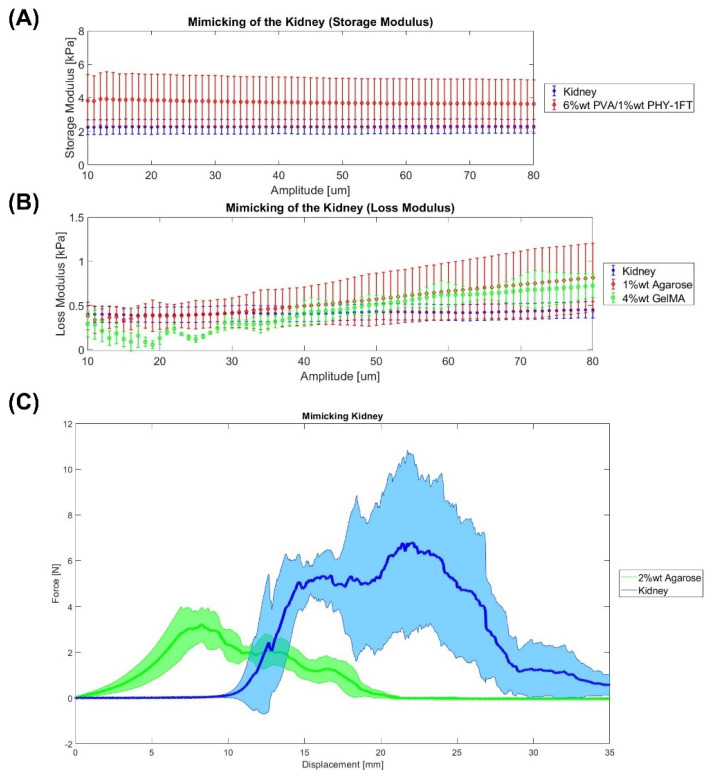
Mimicking the kidney. (**A**,**B**) DMA results. (**C**) Warner–Bratzler shear test results. Data were represented as mean ± SEM values. Each sample has an *n* = 6.

Firstly, the kidney Shore hardness is 36 ± 10 Shore 00, which is the hardest soft tissue measured, and the materials which matched more closely are 2% wt agarose (37 ± 5 Shore 00) and 4% wt GelMA (32 ± 4 Shore 00). Secondly, a kidney tissue storage and loss modulus of 2.38 ± 0.43 kPa and 0.40 ± 0.08 kPa were obtained, respectively. Then, Figure 3A,B show the best materials for mimicking the viscoelastic properties of the kidney. In both storage and loss modulus, different materials appear to be the best option. On the one hand, the storage modulus of 6% wt PVA/1% wt PHY-1FT makes it the best material to mimic the elastic part of the kidney. On the other hand, in terms of the loss modulus part vicious, the closest materials are 1% wt agarose as well as 4% wt GelMA. Concerning the Warner–Bratzler shear test, Figure 3C shows that the best material is 2% wt agarose. Finally, Adams et al. [9] 3D printed different kidney models using the molding technique. The kidney models were made of silicone elastomer, agarose gel, and PDMS. For example, the agarose gel we developed would be an option.

A statistical analysis was carried out to clarify the effectiveness of the present tissue-mimicking analysis. Table 3 shows the statistical analysis of the kidney and the closest materials using the *t*-test. If the *p*-value is higher than 0.05, the null hypothesis stated before is not rejected and the material matches the organ.

### 2.4. Brain

**Figure 4:**
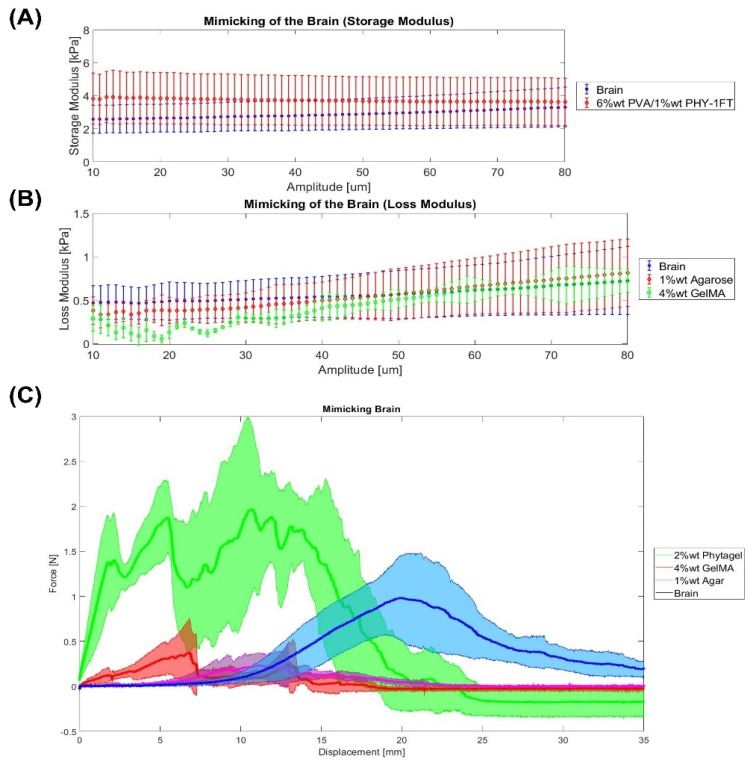
Mimicking the brain. (**A**,**B**) DMA results. (**C**) Warner–Bratzler shear test results. Data were represented as mean ± SEM values. Each sample has an *n* = 6.

Firstly, the brain Shore hardness is 4.5 ± 1.5 Shore 00, which matches the hardness of 2% wt Phytagel (8 ± 2 Shore 00) more closely. Secondly, a brain tissue storage and loss modulus of 2.6 ± 0.84 kPa and 0.47 ± 0.19 kPa were measured, respectively. Then, Figure 4A,B show the best materials for mimicking the viscoelastic properties of the brain. In both storage and loss modulus, different materials appear to be the best option. On the one hand, the storage modulus of 6% wt PVA/1% wt PHY-1FT is the best material to mimic the elastic part of the brain. On the other hand, in terms of the loss modulus part (vicious part), the closest materials are 1% wt agarose and 4% wt GelMA. Then, Dister et al. [23] measured similar viscoelastic properties of the brain tissue by developing a 0.5% wt alginate/0.5% wt gelatine. Concerning the Warner–Bratzler shear test, Figure 4C shows that there are several options: 1% wt agarose, 4% wt GelMA, and 2% wt PHY. Finally, Forte et al. [18] mimicked the brain tissue by developing a composite hydrogel (6% wt PVA/0.85% wt PHY), which then was used for manufacturing a phantom.

A statistical analysis was carried out to clarify the effectiveness of the present tissue-mimicking analysis. Table 4 shows the statistical analysis of the brain and the closest materials using the *t*-test. If the *p*-value is higher than 0.05, the null hypothesis stated before is not rejected and the material matches the organ.

### 2.5. Qualitative Summary

According to the previous results, Table 5 summarizes all values of the tissue-mimicking study.

## 3. Conclusions

In the present work, different materials were tested regarding their viscoelastic behavior, and hardness, as well as their non-linear elastic mechanical response.

All in all, it was seen that the mimicking of soft living tissues is a difficult task, due to the high complexity of organs. Most of them are composed of different tissues that play a key role in terms of structure and mechanical behavior.

According to all the mimicking results that are summarized in the qualitative summary (see Table 5) and statistics, the following are the best materials for mimicking each organ: (1) to mimic the liver, the best materials are 1% wt agarose and CH-1FT; (2) to mimic the heart, the best material is 2% wt agarose; (3) to mimic the kidney, the best material is 4% wt GelMA; and (4) to mimic the brain, the best material is 4% wt GelMA and 1% wt agarose.

Among the different materials, hydrogels are an option for the molding technique, since they offer a good consistency. Additionally, they are soft as well as mostly transparent. In this way, the implications of the current research are interesting for the manufacture of phantoms to be used in medical imaging, preoperative surgical planning in hospitals by doctors, etc.

There is still a lot of work to do in the present field, but this is an excellent starting point for continuing with future research studies in the mimicking of soft living tissues.

## 4. Materials and Methods

### 4.1. Biological Tissue Sample Preparation

Lamb organ (liver, heart, brain, and pancreas) specimens were procured from a local supplier within 24 hours’ post-mortem. These organs were chosen for three different reasons: (1) they cover a high range of different mechanical properties; (2) surgeons often need to accomplish complex surgical tasks in these organs (for example, with the liver a hepatectomy for the tumor removal or very delicate operations in the heart or brain); and (3) there is a lack of knowledge in the mimicking of these four. On the other hand, for the DMA and Warner–Bratzler shear testing, the biological tissues (*n* = 6) were cut using a biopsy punch (16 mm in diameter) to get cylindrical samples: 16 mm diameter and 8 mm height. Regarding the Shore hardness (*n* = 6), no sample preparation was needed as it was directly measured on the tissue’s surface.

### 4.2. Hydrogels Sample Preparation

The hydrogels that were synthetized are: 1% wt and 2% wt agarose gels, 4% wt GelMA, 2% wt PHY, and 6% wt PVA/ 1% wt PHY with one or two freeze-thaw -FT- cycles. These materials were chosen because of their softness. For the DMA and Warner–Bratzler shear testing, the materials’ samples (*n* = 6) were cut using a biopsy punch (16 mm in diameter) to get cylindrical samples: 16 mm diameter and 8 mm height. Regarding the Shore hardness (*n* = 6), no sample preparation was needed.

#### 4.2.1. Agarose Gels

Agarose is a linear polymer with a molecular weight of about 120,000, consisting of alternating D-galactose and 3,6-anhydro-L-galactopyranose linked by α-(1→3) and β-(1→4) glycosidic bonds [43]. The 1% wt and 2% wt agarose gels were produced by mixing deionized water and agarose powder (supplied by Químics Dalmau, Barcelona, Spain). The 1% wt and 2% wt agarose powder amounts were added to the deionized water and magnetically stirred and heated at 90 °C until fully mixed.

#### 4.2.2. GelMA

Gelatine consists of a large number of glycine, proline, and 4-hydroxy proline residues [44]. Methacrylate gelatine (GelMA) was synthesized following a previously described protocol [44]. In short, type A porcine skin gelatin (Sigma Aldrich, San Lui, USA) was mixed at 10% (*w/v*) into phosphate-buffered saline (PBS) at 60 °C and stirred until fully dissolved. Methacrylic anhydride was added at a rate of 0.5 mL/min to the gelatin solution under stirred conditions at 50 °C and allowed to react for 1 h. Following a 5 dilution with additional warm (40 °C) PBS to stop the reaction, the mixture was dialyzed against distilled water using 12–14 kDa cutoff dialysis tubing for 1 week at 40 °C. The solution was lyophilized to generate a porous white foam. GelMA was dissolved into deionized water at 4% (*w/v*) at 37 °C, and Irgacure 2959^®^ (BASF, Mannheim, Germany) was used as a photoinitiator at 0.7% *w/v*. The photoinitiator was dissolved into ethanol absolute (1:2 *w/v*), and then added to GelMA solution. To crosslink GelMA, UV light (365 nm, RegenHU, Villaz-Saint-Pierre, Switzerland) was used.

#### 4.2.3. PHY Gels

Phytagel is produced from a bacterial substrate that is composed of rhamnose, glucuronic acid, and glucose. This polymer is composed of repeating tetrasaccharide units that will form a gel in the presence of mono- or divalent cations [45]. A 2% wt PHY solution was prepared by mixing deionized water and PHY powder, supplied by Sigma Aldrich, San Luis, AR, USA. A 2% wt PHY powder was added to the deionized water and magnetically stirred and heated at 90 °C for 1 h until fully mixed. Then, the solution could cool down.

#### 4.2.4. PVA/PHY Composite Hydrogel (CH)

**Figure 5:**
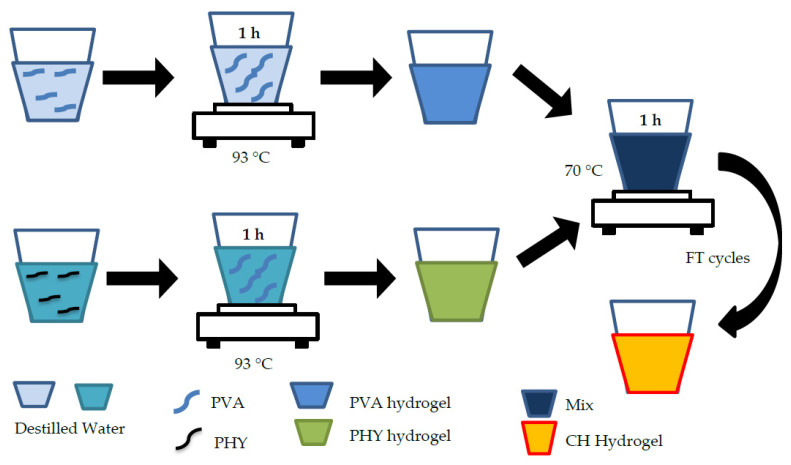
PVA/PHY hydrogel synthesis process.

The composite hydrogel (CH) was produced by mixing PVA (molecular weight 85–124 Da) and PHY, both supplied by Sigma Aldrich, San Luism USA. See Figure 5. PVA is an atactic material that is composed of the 1,3-diol linkages, some of them occur depending on the conditions for the polymerization of the vinyl ester precursor [46]. Solutions were prepared separately with the corresponding amount of powder and deionized water as described in Tan et al. [46]. Amounts of 6% wt PVA and 1% wt PHY powder amounts were added to the deionized water and magnetically stirred and heated at 93 °C for 1 h. Then, when the particles and deionized water were mixed, the solutions could cool down. Afterward, the separate solutions were combined at a 1:1 weight ratio and stirred at 70 °C for 1h. Finally, the samples were physically cross-linked by undergoing one or two freeze-thaw (FT) cycles (24 h) of −18 °C for 16 h, and then the samples were thawed at room temperature for 8 h. 

### 4.3. Parameters for the Mimicking of Soft Tissues

#### 4.3.1. Dynamic Mechanical Analysis (DMA)

DMA is a technique that applies an oscillating force to a material/tissue sample and analyzes the response of the sample to that force [47]. The samples were tested using a DMA Q800 equipment of TA Instruments at 37 °C, 1 Hz and a pre-load force of 0.001 N. DMA in compression calculate the storage modulus (E′), which is the elastic part of the sample; and the loss modulus (E″), which is the vicious part of the sample.

#### 4.3.2. Shore Hardness Test

Shore hardness is a measurement of the resistance of a sample to indentation. There are different scales based on ASTM D2240 testing standards [48]: A, B, C, D, DO, E, M, O, OO, OOO, OOO-S, and R. Each scale results in having values between 0 and 100, where higher values indicate that a sample is harder. The shore is a key parameter for the mimicking of soft living tissues because it measures the consistency of the samples. This is an important aspect of the perception of surgeons.

STM D2240-Durometer Hardness method was used [48]. For that, Shore Durometer Type 00 and 000, supplied by Baxlo, Instrumentos de Medida y Precisión, S.L., Barcelona, Spain, were used for measuring the hardness of the biological tissues (different measurements were done at different parts of the anatomical structure) and material samples. Shore Durometer Type A was also used, but only showed values in the heart.

#### 4.3.3. Warner–Bratzler Shear Test

**Figure 6:**
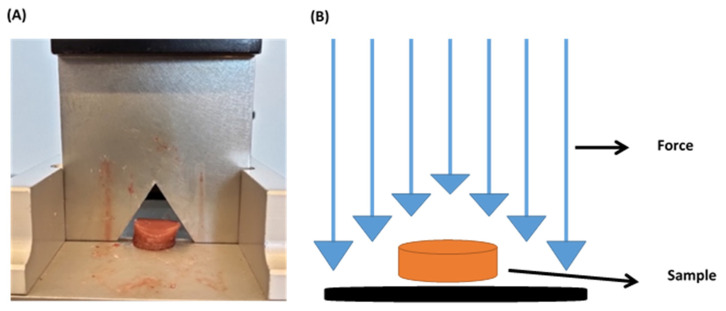
(**A**) Liver sample ready for being cut using the Warner–Bratzler. (**B**) Warner–Bratzler shear test method.

Warner–Bratzler shear test is commonly used in the food industry as a standard characterization method. For example, it has been used to determine the best meat tenderness (toughness) for various types of meat. The Warner–Bratzler consists of a steel frame which is supporting a triangular shear blade (see Figure 6A).

The analysis of the Warner–Bratzler shear test was carried out by focusing on four different parameters: (1) breaking force is the force peak where the cut starts (it is either before the curve is starting to flatten and reaching the maximum force or when there is a change in the curve like a small hole); (2) maximum cutting force is the maximum force of the plot when the sample is being cut; (3) adjustment area is the area under curve until the braking force, and (4) cutting area is the area under the curve from the breaking force until the end.

For creating a tissue-mimicking material for surgical training, the Warner–Bratzler shear test was carried out. This technique is related to the surgeon’s cut feeling operating. A texturometer Texture Analyser TA.XT.plus (Stable Micro Systems, Surrey, UK) was used with a 50 N load cell (Figure 2A). Maximum shear force (N) and area under the curve (J) were measured using the Warner–Bratzler probe. The speed is 1 mm/s during 35 mm of cut. The height of the sample was measured with a digital micrometer.

### 4.4. Statistical Analysis

Statistics were performed using MATLAB R20. Organs mimicking using different materials was assessed using paired sample *t*-test to compare if the material can mimic the organ by focusing on the parameters of the Warner–Bratzler Shear test (maximum force), DMA, and Shore hardness. Data are represented as mean ± SEM (Standard Error of the Mean). *p* ≤ 0.05 (*), *p* ≤ 0.01(**), and *p* ≤ 0.001 (***). The null hypothesis states that an organ and a material are equal. If the *p*-value is lower than 0.05, the hypothesis is rejected; and consequently, it is confirmed that the material cannot mimic the organ. This analysis was only carried out with the most similar materials that can be seen in Figure 1, Figure 2, Figure 3 and Figure 4.

## Figures and Tables

**Table 1:** Statistical analysis of the liver (*p*-value). SH: Shore Hardness. E′: Storage Elastic Modulus. E″: Loss Elastic Modulus. WB: Warner–Bratzler shear test. X states that this material is not able to mimic the tissue and, that is why no statistical analysis is carried out.

	SH	E′	E″	WB
1% wt agarose	0.044	X	0.069	X
2% wt agarose	X	X	X	X
4% wt GelMA	X	X	0.01	X
2% wt PHY	X	X	X	X
6% wt PVA/1% wt PHY-1FT	X	0.016	0.008	X
6% wt PVA/1% wt PHY-2FT	X	X	X	X

**Table 2:** Statistical analysis of the heart (*p*-value). SH: Shore Hardness. E′: Storage Elastic Modulus. E″: Loss Elastic Modulus. WB: Warner–Bratzler shear test. X states that this material is not able to mimic the tissue and, that is why no statistical analysis is carried out.

	SH	E′	E″	WB
1% wt agarose	0.42	X	X	X
2% wt agarose	X	0.28	0.98	X
4% wt GelMA	X	0.18	X	X
2% wt PHY	X	X	X	X
6% wt PVA/1% wt PHY-1FT	X	X	X	X
6% wt PVA/1% wt PHY-2FT	X	X	X	X

**Table 3:** Statistical analysis of the kidney (*p*-value). SH: Shore Hardness. E′: Storage Elastic Modulus. E″: Loss Elastic Modulus. WB: Warner–Bratzler shear test. X states that this material is not able to mimic the tissue and, that is why no statistical analysis is carried out.

	SH	E′	E″	WB
1% wt agarose	X	X	0.10	X
2% wt agarose	0.59	X	X	0.02
4% wt GelMA	0.42	X	0.03	X
2% wt PHY	X	X	X	X
6% wt PVA/1% wt PHY-1FT	X	0.02	X	X
6% wt PVA/1% wt PHY-2FT	X	X	X	X

**Table 4:** Statistical analysis of the brain (*p*-value). SH: Shore Hardness. E′: Storage Elastic Modulus. E″: Loss Elastic Modulus. WB: Warner–Bratzler shear test. X states that this material is not able to mimic the tissue and, that is why no statistical analysis is carried out.

	SH	E′	E″	WB
1% wt agarose	X	X	0.70	0.02
2% wt agarose	X	X	X	X
4% wt GelMA	X	X	0.61	0.12
2% wt PHY	X	X	X	3.89 × 10^−3^
6% wt PVA/1% wt PHY-1FT	X	0.34	X	X
6% wt PVA/1% wt PHY-2FT	X	X	X	X

**Table 5:** Qualitative summary of the mimicking. SH: shore hardness; DMA (E″); DMA (E″); WB (Warner–Bratzler). ✔ corresponds that a material can mimic a certain organ in terms of a certain property.

	Liver				Heart				Kidney				Brain			
	SH	E′	E″	WB	SH	E′	E″	WB	SH	E′	E″	WB	SH	E′	E″	WB
1% wt Agar	✔		✔		✔						✔				✔	✔
2% wt Agar						✔	✔		✔							
4% wt GelMA			✔			✔			✔		✔				✔	✔
2% wt PHY																✔
6% wt PVA/1% wt PHY-1FT		✔	✔							✔				✔		
6% wt PVA/1% wt PHY-1FT																

## Data Availability

Data can be shared upon request.
